# Influence of the Mixtures of Vegetable Oil and Vitamin E over the Microstructure and Rheology of Organogels

**DOI:** 10.3390/gels8010036

**Published:** 2022-01-05

**Authors:** Renata Miliani Martinez, Pedro Leonidas Oseliero Filho, Barbara Bianca Gerbelli, Wagner Vidal Magalhães, Maria Valéria Robles Velasco, Suzana Caetano da Silva Lannes, Cristiano Luis Pinto de Oliveira, Catarina Rosado, André Rolim Baby

**Affiliations:** 1 Department of Pharmacy, Faculty of Pharmaceutical Sciences, University of São Paulo, São Paulo 05508-000, Brazil; mvrobles@usp.br; 2 Department of Applied Physics, Physics Institute, University of São Paulo, São Paulo 05508-000, Brazil; plof@if.usp.br; 3 Center of Natural and Human Sciences, Federal University of ABC, Santo André 09210-170, Brazil; barbara.gerbelli@ufabc.edu.br; 4 Research & Development Laboratory-Chemyunion Ltd., Sorocaba 18103-065, Brazil; wagner.magalhaes@chemyunion.com; 5 Department of Biochemical and Pharmaceutical Technology, Faculty of Pharmaceutical Sciences, University of São Paulo, São Paulo 05508-000, Brazil; scslan@usp.br; 6 Department of Experimental Physics, Physics Institute, University of São Paulo, São Paulo 05508-000, Brazil; crislpo@if.usp.br; 7 CBIOS—Universidade Lusófona’s Research Center for Biosciences and Health Technologies, 1749-024 Lisbon, Portugal; catarina.rosado@ulusofona.pt

**Keywords:** organogel, candelilla wax, 12-hydroxystearic, vitamin E, biomaterials, rheology, SAXS

## Abstract

Candelilla wax (CW) and 12-hydroxystearic acid (12HSA) are classic solid-fiber-matrix organogelators. Despite the high number of studies using those ingredients in oily systems, there is scarce literature using a mixture of oil and antioxidants. Vitamin E (VE) is an important candidate for its lipophilicity and several applications on pharmaceutical, cosmetics, and food industries. In this work, we investigated the influences of mixtures between vegetable oil (VO) and VE on the microstructures and rheological properties of CW and 12HSA organogels. A weak gel (G′′/G′ > 0.1) with a shear-thinning behavior was observed for all samples. The presence of VE impacted the gel strength and the phase transition temperatures in a dose-dependent pattern. Larger and denser packed crystals were seen for 12HSA samples, while smaller and more dispersed structures were obtained for CW organogels. The results obtained in this work allowed the correlation of the structural and mechanical properties of the organogels, which plays an important role in the physical-chemical characteristics of these materials.

## 1. Introduction

Organogels are tridimensional structures formed by the organization of molecules in order to hold an organic solvent. Gel formation can be investigated by crystal network observation via polarized microscopy, rheological viscoelastic properties, and/or physical structure at the atomic scale via small-angle X-ray scattering (SAXS) [1]. The type of organogelator and the production process allow the classification of organogels into three categories: (a) fluid filled matrix; (b) solid fiber matrix; and (c) chemical organogels [1]. Low-molecular-weight organogelators (LMWOs), such as candelilla wax (CW) and 12-hydroxystearic acid (12HSA), can self-organize within an organic system, forming a thermoreversible organogel [2]. The gelation occurs upon the total melting of organogelators, followed by a cooling process as the driving force for the nucleation of the organogelator molecules. This mechanism, known as solid fiber, leads to a three-dimensional fibrillar network structure that holds the organic phase [1].

The crystallization pattern (the shape, size, and distribution of crystals) of organogels is a manifestation of several interaction forces between organogelators and the organic solvent and is affected by many variables, such as the physical and chemical properties of the components, the organogelator/solvent ratio, the total melting of the organogelator, and the cooling rate [3]. The starting point for organogelification is the nuclei formation that grows into the crystalline network. Heterogeneous nucleation is the most favorable type of process, when the nuclei are formed by a substrate that provides the orientation for the crystalline network. The substrate can be any surface, such as the walls of the container or even impurities present in solutions [4]. Therefore, every organogel component is a potential nucleation agent, especially if it is solid or crystalline by itself. For that reason, most organogel studies are conducted with one simple organic phase for each organogelator, such as sunflower oil and CW [5,6] or canola oil and 12HSA [7]. However, for practical applications, more complex matrices are often used. For instance, when oil is used as the organic phase of organogels, the presence of a lipophilic antioxidant could protect against degradation. Despite robust information regarding the organogel’s structure using mixed sterols [8] and lecithin blended with other materials [9], few information about mixtures with antioxidants is available.

Our research group has been studying the influence of antioxidants on colloidal systems for the past decade and has recently started exploring organogels and bigels with vitamin E (VE) [10]. Despite beneficial to humans, VE is not naturally produced by the human body and must be topically or orally delivered. This antioxidant is involved in cutaneous protective pathways against aging, atopic dermatitis, and melanoma, for instance [11,12,13]. It also plays an important role in food industry as a shelf-life enhancer of oily products or to increase oxidative stability in heated oils [14]. Therefore, it is used in a wide range of concentration into pharmaceutical, cosmetic, and food products, with several applications. Despite the wide use, VE was still not explored in organogel systems. The popularization of organogels to replace trans-fat contents in foods [15] and the benefits over the sensory and transdermal delivery of molecules [16] drive the necessity to understand the effect that additives have over the gel formation. The variation of organogels’ structures may be very detrimental to the formulation development.

In this research work, the influences of mixtures between vegetable oil (VO) and VE on the microstructures and rheological properties of CW and 12HSA organogels were verified. The VE at several concentrations in CW and 12HSA organogels were investigated using polarized microscopy, rheology, and SAXS, providing detailed structural and mechanical information.

## 2. Results and Discussion

**Figure 1:**
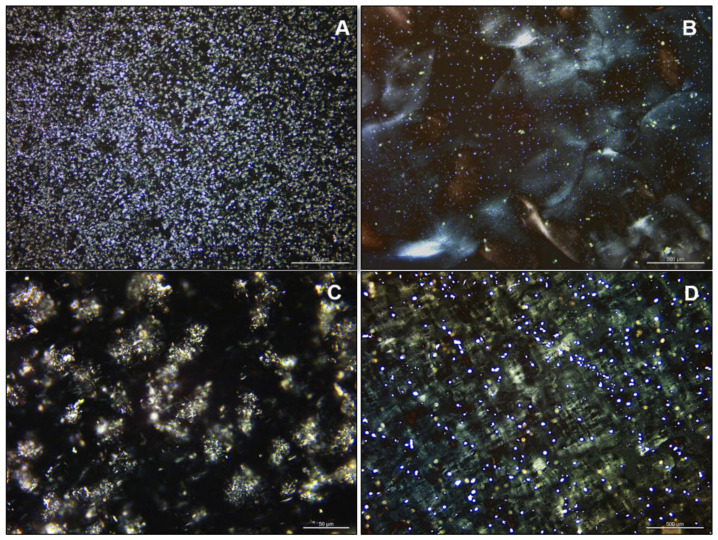
Polarized photomicroscopy images of organogels: (**A**) candelilla wax 2 (CW2):vegetable oil 97 (VO97):vitamin E 1 (VE1); (**B**) 12-hydroxystearic acid 2 (12HSA2):VO96:VE2; (**C**) CW2:VO98; and (**D**) 12HSA2:VO78:VE20. The scale bars are 500 µm in (**A**,**B**,**D**), and the scale bar is 50 µm in (**C**).

The macroscopic evaluation showed rigid and transparent gels with little syneresis for 12HSA organogels, while soft and opaque organogels were observed for CW samples (Figure S1). The polarized microscopy observation (Figure 1) explained those findings, since a heterogeneous and discontinuous crystal distribution was observed for CW organogels (Figure 1A), instead of dense and homogeneous crystals in 12HSA samples (Figure 1B). Similar patterns were observed previously in other organogels with the same concentrations of those organogelators [4,5]. However, for CW organogels, a tighter pattern with more evenly distributed crystals may be seen since the natural origin and the variable composition of the CW [17].

Throughout the concentration range of VE tested (0–20%), the size and distribution of crystals were the same under polarized microscopy. The methodology was not a statistical image analysis; therefore, the rheology and SAXS investigations were used for better understanding the crystallization pattern of the organogels.

**Figure 2:**
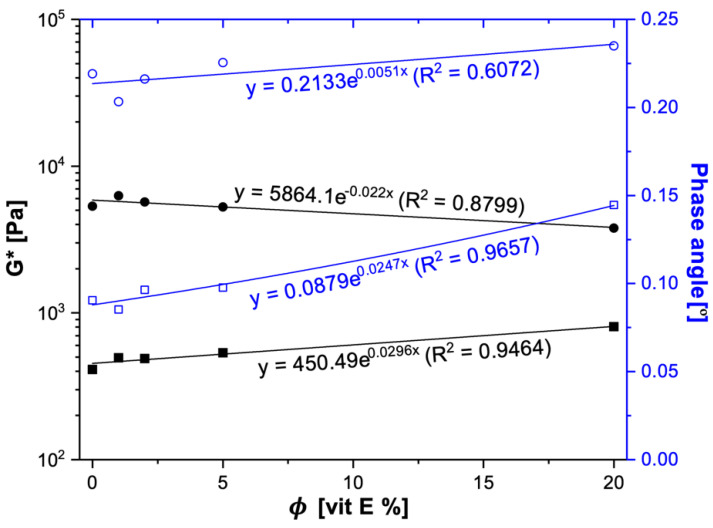
Frequency sweep measurements results for formulations with 12HSA:VO (●) and CW:VO (■) in terms of G* (closed symbols) and the phase angle (open symbols) at 1 Hz. The experimental data are represented by symbols, and the fitting model is represented by lines according to the percentage of VE available (Φ).

For all samples, in the frequency sweep measurements, the elastic moduli (G’) dominated the viscous moduli (G”), i.e., G’ > G”, throughout the range where the applied shear was evaluated (Figure S2), which marked solid-like behaviors of the gels [18]. We investigated the contribution of the VE concentration to the complex modulus (G*)—a combination of the G’, the G”, and the phase angle—at 1 Hz for a comparison [19]. For the CW organogels, the G* increased exponentially with the increasing of the VE content, while the exact opposite effect was observed for the 12HSA organogels. Apparently, the composition of the oily phase (VE and VO) affected the organization of the organogels, increasing the G* values for CW and decreasing the G* values for 12HSA (Figure 2). Higher G* values were related with higher network gel strengths [20]. The phase angle was lower than the unity in the whole range of frequency investigated (Figure 2). Those results contributed to verifying the solid-like behaviors of all organogels [17]. The loss tangent was the tangent of the phase angle denoted as: tanδ = G′′/G′. The organogels containing 12HSA showed values of the loss tangent higher than 0.1 (tanδ > 0.1), typical of the so-called weak gels, throughout the whole range of frequency tested [21]. However, the CW organogels presented an elastic behavior (tan*δ* < 0.1) at lower frequencies (<1 Hz) from 0 to 5% VE concentrations [22]. When higher VE amounts were used (20%), the behavior changed to weak gels (tan*δ* > 0.1). The VE hydrophilic area can impact somehow the arrangement of crystals, as described earlier, in combination with lecithin organogels [23]. In addition, when increasing the VE concentration, we proportionally reduced the amount of VO, which impacted the oily phase composition and could have changed its physical properties for crystal binding.

**Figure 3:**
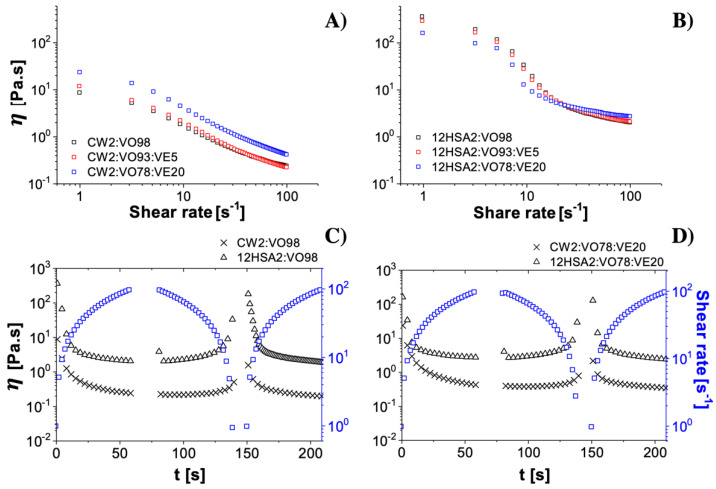
Viscosity (η) versus shear rate for CW organogels (**A**) and for 12HSA organogels (**B**). Structure recovery after shear stress (black dots) for the organogels without VE (**C**) and with VE (**D**).

The flow curve measurements showed a non-Newtonian shear-thinning behavior for all samples, characterized by a decrease in the viscosity as the shear rate increased (Figure 3A,B). The values of the parameters obtained using the Carreau–Yasuda model (Equation (13)) are shown in Table 1 and confirmed the shear-thinning behavior (η < 1). Moreover, because of the smaller relaxation time (λ) values, no restriction of mobility was observed for the crystal network inside the organogels [24]. Closer crystals showed slower relaxation times, as observed for the CW organogels. The shear-thinning property is particularly interesting for applications that require the material to flow under an applied force, such as implants [25], topical products [26], injectable products [27], and foods [28]. The viscosity was higher for 12HSA organogels than that of the CW ones, which corroborated the microscopy findings earlier described. A more densely packed crystal network, such as seen for 12HSA organogels, can reduce the mobility of the components inside the organogels by increasing the tortuosity of the system [29]. Crystals acted as barriers against the organogel flow during the viscosity analysis.

The zero-shear viscosity ($\eta_{0}$) is an important parameter to evaluate the consistency (the initial viscosity when the sample starts to flow). Consistency can be is translated as a physical attribute of the first contact between skin and formulation for topical products [30], stability [31], and package design [32]. For all compositions, an initial plateau was observed followed by a large drop in viscosity (shear-thinning region) and finally a second plateau (infinite shear viscosity). This behavior is characteristic of shear-thinning fluids, and it was more accentuated for 12HSA organogels (Figure 3B). The composition of the oily phase affected the consistency of the samples. When higher amounts of VE (and proportionally lower amounts of VO) were used, we observed an increase in the consistency for CW organogels and a decrease in the consistency for 12HSA organogels. However, the 12HSA organogels showed naturally much higher $\eta_{0}$ values. Along with the G* results, the consistency suggested that the composition of the oily phase affected crystal organization into the organogels. Earlier studies showed that the 12HSA network can be disrupted when lecithin is used in association as a co-oleogelator [33]. Likewise, lecithin showed a synergic effect with candelilla and other natural waxes on oleogels formation [15]. In our case, VE played a role as a co-organogelator, along with the reduction of the VO availability. Less oil could hinder the fibrous network for 12HSA, while alleviating the necessity of oil support for the CW network. Another hypothesis is that the combination of VE and VO presents singular physical-chemical properties for organogelation.

The time-dependency behavior and the structure recovery after stress are the two phenomenon involved with thixotropic fluids [34]. Thixotropy plays an important role in the sensory and stability features of topical and oral formulations. It is related with the spreadability of topical formulations over skin [35] and to predict the deformation of foods during the processing and handling operations [36]. The flow curves showed a recovery in the 12HSA viscosity after shearing at the same extend, despite the alterations of the oily phase. However, no recovery was observed for the CW organogels (Figure 3C,D). When a shear force is applied over oleogels, the ability to retain oil decreases since small crystals are formed [29]. In our experiment, the shear applied over CW organogels, which naturally showed a small crystals distribution, probably promoted an oil migration and increased the difficulty in recovering the original structure.

**Figure 4:**
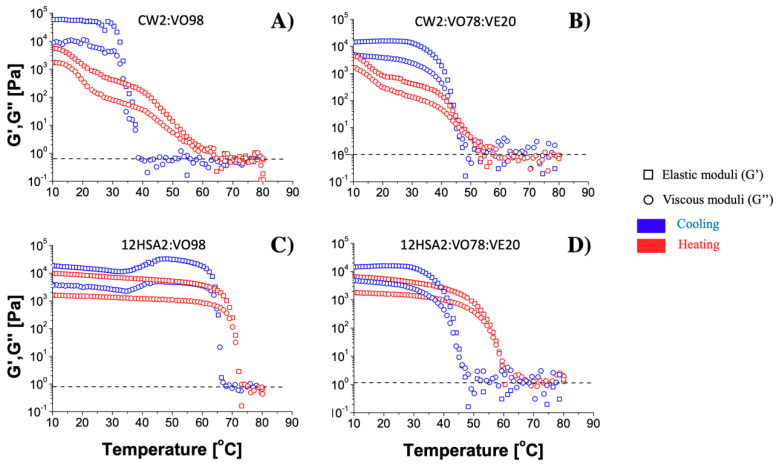
Temperature ramp test results for CW2:VO98 (**A**), CW2:VO78:VE20 (**B**), 12HSA2:VO98 (**C**), and 12HSA2:VO78:VE20 (**D**) organogels. In Table 1, the organogel melting temperature and the gelation transition temperature obtained from the ramps where G′≈G″ are presented.

**Figure 5:**
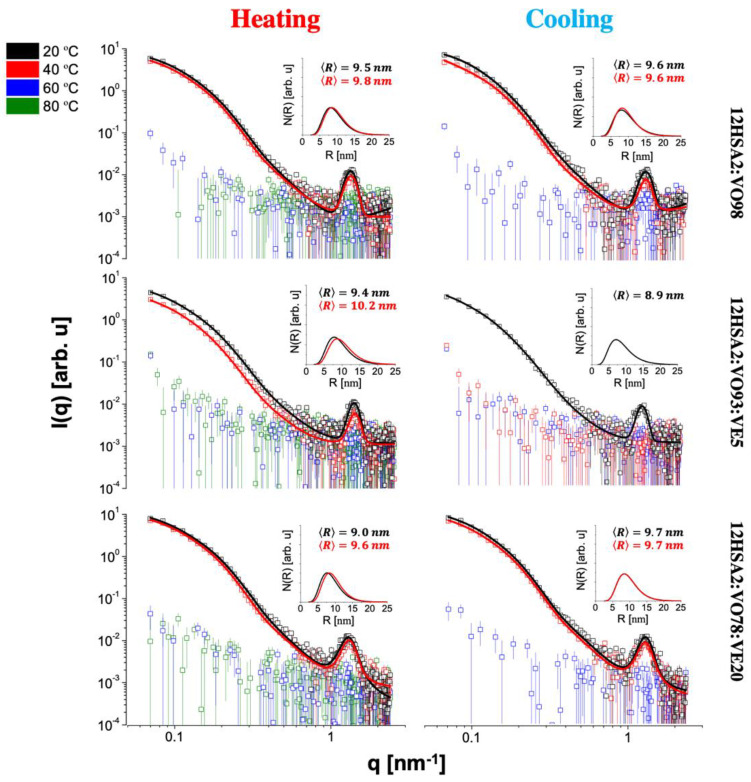
Small-angle X-ray scattering (SAXS) data for the 12-HSA organogels on heating and on cooling (open symbols) and fitted with Equation (12) (continuous lines). The insets correspond to the cylinder radius size distributions. The average radius for each distribution is shown as well.

The thermal behavior of the gel–sol or sol–gel phase transition domains (PTDs) of organogels was evaluated using a temperature ramp test [37]. All PTDs shifted to smaller temperatures proportionally to the addition of VE (Figure 4 and Figure 5). However, the shift was more accentuated for the 12HSA organogels. The melting temperature (Tmelt) for the 12HSA organogels without VE was close to 79 °C, as reported by Esposito and coworkers [38] at the same concentration, but higher than 37 °C for the gelation temperature (Tgel). Besides the different rheometer geometries and oily phases, the authors used strategies that could impact the crystallization pattern, such as a slower cooling rate compared to in our experiment (1 °C/min), a surfactant, and a co-solvent [3,37]. They pointed that the addition of the surfactant disrupts the crystalline network, causing the reduction of the gel density, which was similar with our findings for VE. VE also impacts more complex systems, such as nanoemulsions. A decrease in the temperature of fat crystallization in oil-in-water stearin-rich milk fractions was observed for sodium caseinate-stabilized nanoemulsion containing VE [39]. Several works have studied the impact of VE in cellular membrane models using phospholipids. Alpha tocopherol, specifically, works in bilayer membranes’ de-stabilization by forming complexes with lipid components [40]. This mechanism is plausible to explain the reduction of PTD temperatures in our results; nevertheless, the specific interaction between VE and the organogelators is currently unknown. In this sense, another hypothesis is that VE modifies the oily phase properties, consequently impacting the rheological properties of the organogel itself.

For the CW organogels, the pattern of the heating curve showed a gradual melting process, different from the sharp decrease in G’ for 12HSA. CW is a mixture of n-alkanes, esters of acids, alcohols, sterols, and free acids [9]. Each component has its own melting point and impacts the total organogel Tmelt, as well as in the interactions with VE, if they exist. Interestingly, the cooling ramp seemed to organize the crystals, since the cooling rate was controlled (5 °C/min), opposing to the organogel production when it was naturally cooled. This may explain the higher G’ values after the temperature ramp test. Regarding this aspect, the 12HSA samples recovered approximatively their initial G’ values (Figure 4C,D), while the CW organogels increased 10 times the initial G’ magnitude (Figure 4A,B), reassuring a thixotropic effect for the 12HSA organogels but not for the CW organogels. No significant changes were observed when changing the oily phase, except for the discrete reduction of PTD temperatures previously described.

**Figure 6:**
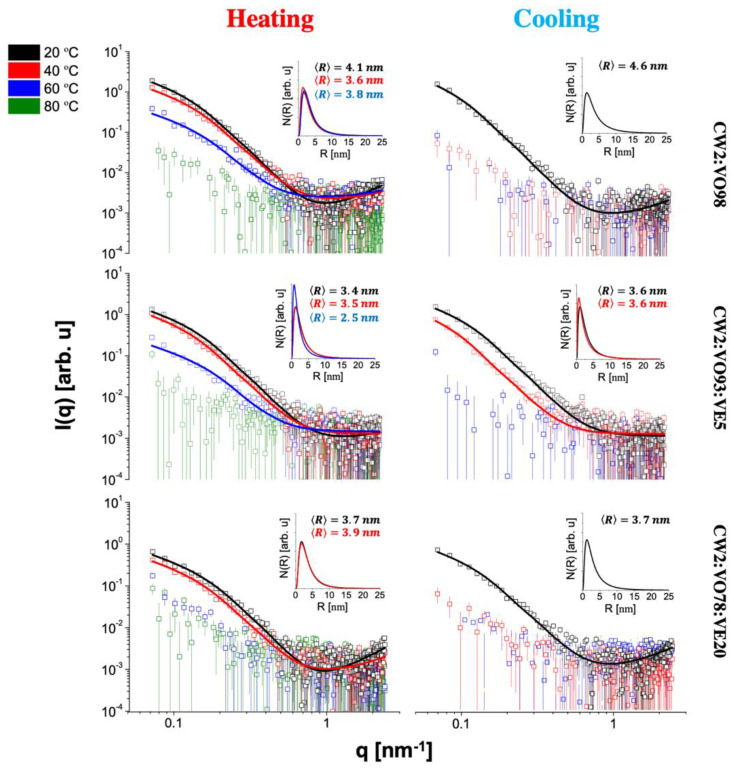
SAXS data for the CW organogels on heating and on cooling (open symbols) and fitted with Equation (12) (continuous lines). The insets correspond to the cylinder radius size distributions. The average radius for each distribution is shown as well.

To better understand the effect of the oily phase over the network structure at a nanoscale, SAXS experiments were performed, which brought important information about the shape and size of the gel nanostructure [41]. The SAXS data for the 12HSA and CW organogels (open symbols) at all investigated temperatures for heating and on cooling are shown in Figure 5 and Figure 6, respectively. In the studied length scale, the profiles of the curves obtained for all organogelators were different, suggesting nanostructures with a distinct size and/or shape. A peak at q of approximately 1.3 nm^−1^ was observed for the 12HSA organogels and was attributed to the (001) Bragg reflection of the 12HSA crystal [42]. It is interesting to note that even CW also has a crystalline structure according to the X-ray diffraction data from previous works [43,44], which was not observed in our data. Possibly, its neatly ordered molecular arrangement was disrupted in the organogel formation, since its main component hentriacontane (~79%, *w*/*w*) is highly soluble in organic solvents, such as VOs [5].

**Figure 7:**
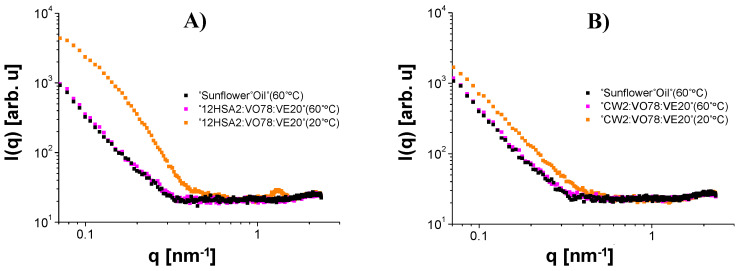
Comparison between the SAXS curves of sunflower oil at 60 °C, the major component of the organogels presented in this manuscript, and the samples containing 12HSA (**A**) and CW (**B**), at 60 and 20 °C. In this example, the curves collected at 60 °C are almost perfectly overlapped. This fact makes some of the treated curves presented in Figure 5 and Figure 6 very noisy in all q values. For the other curves, like the ones collected at 20 °C, only their high q regions are noisy.

On heating, a structural gel-to-sol transition was clearly seen for all materials. After this event, the sample scattering curve practically coincided with the sunflower oil curve (Figure 7), the major component used in the sample preparation, and the data reduction led to very noisy curves. This fact made it easier to identify, in Figure 5 and Figure 6, the mentioned transition, which occurred at T≥40 °C for all 12HSA compositions and CW2:VO78:VE20 and only at T≥60 °C for all CW compositions, except CW2:VO78:VE20, in agreement with the temperature ramp tests. Furthermore, the CW organogels seemed to be more thermal-resistant than the ones composed of 12HSA, in the sense that they kept their nanostructures at high temperatures up to, at least, 60 °C, except CW2:VO78:VE20. For this particular composition, it was unclear if the melting of the gel at lower temperatures (relative to the other compositions) was caused by a specific effect of VE over the organogel structure or if it was a direct result of change in the oily phase properties due to the addition of VE followed by the reduction of VO. Nevertheless, from all these observations, one can argue that the sol–gel transition is composition-dependent.

On cooling, the sol-to-gel transition was, in general, distinct from the one observed on heating, except for 12HSA2:VO98 and 12HSA2:VO78:VE20. Gels were formed at T≤60 °C for all 12HSA compositions (except 12HSA2:VO93:VE5) and only at T≤20 °C for all CW organogels (except CW2:VO93:VE5). The combination 93% VO + 5% VE seemed odd in both types of materials: with 12HSA, the gel formation was difficult, whereas with CW the exact opposite occurred. This fact corroborated once more that the particular composition of the gel had a great influence on its properties, including the sol-to-gel transition temperature. It is quite interesting to observe that the materials containing CW were less prompt to form gels on cooling, whereas the same gels on heating lasted longer at more high temperatures than the ones composed of 12HSA, as discussed before.

Aiming to retrieve quantitative information on the organogel nanostructure, the SAXS data were satisfactorily fitted using Equation (13) (continuous lines of Figure 5 and Figure 6). From the fittings, the average radius, 〈R〉, and the size distributions of R values (graphs insets in Figure 5 and Figure 6) were obtained. For the 12HSA organogels, 〈R〉 was approximately 10 nm, in agreement with the value reported for 12HSA gel in toluene [45], whereas for the CW materials, 〈R〉 was approximately 4 nm. This difference in 〈R〉 might be directly associated to the differences observed in the polarized microscopy results. With the temperature increase, 〈R〉 slightly increased for all compositions containing 12 HSA, whereas there was no apparent pattern for the CW organogels. In general, 〈R〉 values on cooling were different compared to the ones obtained on heating. This sort of hysteresis was also observed in the measured rheological properties. Regarding the size distributions, they were positively skewed, i.e., the distributions had more data on the right tail. A dispersion of R values around 30% was found in all cases, which was ~1.5 times higher than the value found for 12HSA gel in toluene [45], likely related to the presence of sunflower oil. To the best of our knowledge, there is no reference value for CW organogels, and this is the first time that this kind of SAXS analysis is applied to this material. All in all, the SAXS data for both types of organogel pointed to the structural differences between them, which were satisfactorily quantified by the use of an advanced analysis. Moreover, the gel-to-sol transition on heating and the sol-to-gel transition on cooling were quite evident in the presented curves and strongly dependent on the composition of each investigated organogel.

### SAXS Model

SAXS data were modeled assuming non-interacting randomly oriented cylindrical particles with radius R and length L. In this case, the theoretical scattering intensity is given by:

(1) I(q)=sc⋅P(q,R,L)+back,

where the parameter sc is a scale factor and P(q,R,L) is the cylinder normalized form factor [46]:

(2) $$P\left(q,R,L\right)=\int_{0}^{\frac{\pi }{2}}{\left[\frac{2J_{1}\left(qR\sin\alpha \right)}{qR\sin\alpha }\frac{\sin\left(0.5qL\cos\alpha \right)}{0.5qL\cos\alpha }\;\right]}^{2}\sin\alpha d\alpha ,$$

where $J_{1}\left(x\right)$ is the first-order Bessel function and α is the angle between the axis of the cylinder and the scattering vector $\vec{q}$. In most of the samples was observed a non-flat curve at high q values, related to a gel–buffer mismatch in data reduction. To compensate this effect, a non-linear background, back, was introduced. In our case, a simple second-order polynomial function satisfactorily fit this region of the SAXS curves.

Under the hypothesis, the cylinders are sufficiently long (L≫R), used in previous works [45]. Equation (2) can be approximated as a product of the longitudinal factor, $P_{rod}\left(q,L\right)$, parallel to the cylinder axis, and the scattering cross-section function, $P_{CS}\left(q,R\right)$ [47]:

(3) $$P\left(q,R,L\right)=P_{rod}\left(q,L\right)P_{CS}\left(q,R\right),$$

where

(4) $$P_{rod}\left(q,L\right)=\frac{Si\left(q\cdot L\right)}{0.5\cdot qL}-{\left[\frac{\sin\left(0.5\cdot q\cdot L\right)}{0.5\cdot q\cdot L}\right]}^{2},$$

(5) $$Si\left(x\right)=\int_{0}^{x}\frac{\sin t}{t}dt,$$

(6) $$P_{CS}\left(q,R\right)={\left[A_{CS}\left(q,R\right)\right]}^{2},$$

(7) $$A_{CS}\left(q,R\right)=\frac{2\cdot J_{1}\left(q\cdot R\right)}{q\cdot R}.$$

In this study, after several tests, L was fixed at 200 nm, which provided the best fittings and fulfilled the above hypothesis regarding long cylinders. Taking the R polydispersity into account, the normalized form factor was written as [48]:

(8) $$\langle P(q,R,L)\rangle =\frac{\int_{-\infty }^{\infty }N\left(R\right)V{\left(R,L\right)}^{2}P\left(q,R,L\right)dR}{\int_{-\infty }^{\infty }N\left(R\right)V{\left(R,L\right)}^{2}dR},$$

where P(q,R,L) is defined by Equation (2) or Equation (3) and $V\left(R,L\right)=\pi R^{2}L$ is the cylinder volume. In this study, N(R) was a log-normal distribution [49,50]:

(9) $$N\left(R\right)=\frac{1}{R\omega \sqrt{2\pi }}\exp\left(-\frac{1}{2}{\left(\frac{\ln R-\theta }{\omega }\right)}^{2}\right),$$

where θ and ω are the average and the standard deviation, respectively, of a Gauss distribution of lnR. The expected (average) value for the variable R and its standard deviation were described as [49,50]:

(10) $$\mu =\exp\left(\theta +\frac{\omega^{2}}{2}\right),$$

(11) $$\sigma =\sqrt{\exp\left(2\theta +\omega^{2}\right)\cdot \left(\exp\left(\omega^{2}\right)-1\right)}.$$

Therefore, rewriting Equation (1), the final fitting equation was expressed as:

(12) I(q)=sc⋅〈P(q,R,L)〉+back.

For the organogels containing 12HSA, its crystal structure contributed with a peak at q of approximately 1.3 nm^−1^ (see Figure 5). This feature was taken into account by introducing a Gauss function term in Equation (1), as performed in previous works [45].

## 3. Conclusions

The design of the organogel’s composition plays an important role in the physical-chemical properties of materials. We found distinct structures for all organogels’ compositions. While 12HSA built a highly packed network, CW showed sparse crystals observed with polarized microscopy. All organogels were characterized as weak gels with a shear-thinning behavior, but only 12HSA organogels showed thixotropy. The gradual replacement of VO by VE in the oily phase showed an improvement of the gel strength for CW, but a reduction for 12HSA organogels. Likewise, all phase transition temperatures were reduced in a dose-dependent pattern, especially for 12HSA. Larger crystals that slightly increased upon heating were observed via SAXS for 12HSA when compared with for CW. Phase transition was strongly dependent on the composition of the organogel. Further investigation regarding the deeper structure of organogels could clarify whether VO and VE form a new oily phase with different properties or if VE and organogelators compete for VO availability. Those data would be especially beneficial for pharmaceutical, cosmetics, and food exploitation of organogels.

## 4. Materials and Methods

### 4.1. Organogel Preparation

Sunflower (Helianthus annuus) oil (VO) with a high oleic content, was purchased from Agri Pure 80, Cargill Agrícola S/A, São Paulo, SP, Brazil. The organogelators CW and 12HSA were purchased from Double Refined Candelilla Wax 102P, Koster Keunen Inc, Wartertown, CT, USA, and A. Azevedo, São Paulo, SP, Brazil, respectively. VE (dl-α-Tocopherol) was purchased from DSM, Parsippany, NJ, USA. All materials were used as received. The production of organogels consisted in the mixing of VO with CW or 12HSA at 85 °C upon continuously stirring at 200 rpm (RW 20; IKA-Werke, Staufen, Germany), followed by the addition of VE once the organogelator was completely melted. The component amounts of each sample are shown in Table 2. At this point, the mixing was continued for 5 min, followed by rest and cooling at room temperature for 24 h. The batch sizes were standardized at 50 g to insure the same thermal heating transfer characteristics.

### 4.2. Microscopy Tests

Polarized microscopy was performed in a DM2700, Leica Microsystems, Wetzlar, Germany, with a 40× objective (Leica HI Plan 40×/0.65 POL). The images were captured with a digital camera Leica MC120 HD (Leica Microsystems, Wetzlar, Germany) and analyzed with the LEICA Application Suite Software (Leica, Wetzlar, Germany). All samples were visualized over glass plates without dilution.

### 4.3. Rheological Characterization

Rheological measurements were performed in a strain rheometer (TA Instruments, DHR-2, New Castle, DE, USA) with the crosshatched parallel plates with a 20 mm geometry (gap: 300.0 ± 0.1 µm) coupled to a Peltier system for temperature control. Approximately 0.5 g of organogels was transferred to the geometry and left still for 3 min to the equilibrium temperature of 25 °C before each measurement. We conducted 3 different experiments in order to evaluate the elastic modulus (G’) and the viscous modulus (G”) behavior when submitted to stress: frequency sweep tests, flow curve tests, and temperature ramp tests. All measurements were conducted in triplicates. Frequency sweep tests were carried out in the range of 0.1–100 rad/s at 25 °C in the viscoelastic linear region previous evaluated by the amplitude sweep test for each sample. We monitored the G’, the G”, and the phase angle (ᵟ). Three flow curve tests were performed in controlled stress conditions: increasing the stress from 0 up to 100 s^−1^, decreasing from 100 to 0 s^−1^, and increasing again from 0 to 100 s^−1^. Intervals of 60 s were adopted between every curve at 25 °C. We monitored the viscosity (η) behavior during shear stress. The Carreau–Yasuda viscosity model [51] was used to fit the flow curves:

(13) $$\eta \left(\gamma \right)-\eta_{\infty }=\left(\eta_{0}-\eta_{\infty }\right){\left[1+{\left(\lambda \gamma \right)}^{a}\right]}^{\frac{\left(n-1\right)}{a}},$$

where η is the shear-dependent viscosity, η_0_ is the zero-shear viscosity, η_∞_ is the infinite shear viscosity, λ is the relaxation time, a is a parameter describing the rate of the transition from the Newtonian plateau to the power law region, and n is the power law index.

Dynamic temperature ramp measurements were performed at 1 Hz in the linear viscoelastic regime, ranging from 10 to 70 °C and from 70 to 10 °C, with intervals of 60 s between curves in a ramp rate of 5 °C/min.

### 4.4. SAXS

SAXS measurements were performed using a Nanostar (Bruker) instrument equipped with a microfocus Genix 3D system (Xenocs). The samples were maintained in quartz capillaries with a mean diameter of 1.5 mm, and the scattered intensity was collected with a 2D Vantec-2000 detector. The sample-to-detector distance was ~1 m, which provided an effective range of the modulus of the transfer moment vector q experimentally accessible of 0.08–2.3 nm^−1^, with q = 4π sin(θ)/λs, where 2θ is the scattering angle and λs = 0.154 nm is the X-ray wavelength from the copper Kα radiation). The sample temperature was varied using a circulating water bath, with a precision of 0.1 °C. An equilibration time of 600 s was applied, after the samples reached one of the selected temperatures (20, 40, 60, and 80 °C). For the treatment of the 1D data, obtained through the azimuthal integration of the 2D data, the SUPERSAXS package [52] was used and consisted of normalization by the measuring time (1800 s) and sample transmission, followed by the subtraction of the blank scattering. The scattering from the sunflower oil, measured at the same temperatures of the samples, was taken as the blank, since it is the major component of these organogels.

## Figures and Tables

**Table 1:** Fitting parameters of the Carreau–Yasuda equation for flow curves and approximate phase transition temperatures Tmelt (melting temperature) and Tgel (gelation temperature).

Code	$\eta_{0}$ (Pa·s)	$\eta_{\infty }$ (Pa·s)	λ (s)	*n*	a	Tmelt (°C)	Tgel (°C)
CW2:VO98	9.37 ± 0.03	0.15 ± 0.01	0.39 ± 0.01	−0.26 ± 0.01	2.15 ± 0.03	55	34
CW2:VO93:VE5	16.06 ± 1.26	0.14 ± 0.01	0.38 ± 0.02	−0.44 ± 0.33	1.24 ± 0.03	55	38
CW2:VO78:VE20	24.93 ± 0.18	0.22 ± 0.01	0.41 ± 0.01	−0.29 ± 0.02	2.49 ± 0.04	50	39
12HSA2:VO98	350.08 ± 8.80	2.19 ± 0.01	0.27 ± 0.01	−1.65 ± 0.01	3.01 ± 0.01	72	66
12HSA2:VO93:VE5	284.41 ± 7.25	2.21 ± 0.01	0.29 ± 0.01	−1.40 ± 0.01	3.63 ± 0.01	63	58
12HSA2:VO78:VE20	150.89 ± 4.09	2.94 ± 0.01	0.29 ± 0.01	−1.39 ± 0.01	4.82 ± 0.01	59	46

**Table 2:** Organogels studied in this work. The acronyms “CW”, “VO”, “12HSA”, and “VE” mean candelilla wax, vegetable (sunflower) oil, 12-hydroxystearic acid, and vitamin E, respectively.

Code	% CW (*w*/*w*)	% 12HSA (*w*/*w*)	% VO (*w*/*w*)	% MO (*w*/*w*)	% VE (*w*/*w*)
CW2:VO98	2.0	-	98.0	-	-
CW2:VO97:VE1	2.0	-	97.0	-	1.0
CW2:VO96:VE2	2.0	-	96.0	-	2.0
CW2:VO93:VE5	2.0	-	93.0	-	5.0
CW2:VO78:VE20	2.0	-	78.0	-	20.0
12HSA2:VO98	-	2.0	98.0	-	-
12HSA2:VO97:VE1	-	2.0	97.0	-	1.0
12HSA2:VO96:VE2	-	2.0	96.0	-	2.0
12HSA2:VO93:VE5	-	2.0	93.0	-	5.0
12HSA2:VO78:VE20	-	2.0	78.0	-	20.0

## Supplementary Materials

The following are available online at https://www.mdpi.com/article/10.3390/gels8010036/s1, Figure S1: Macroscopic aspecst of organogels: (A) 12HSA2:VO98; (B) 12HSA2:VO78:VE20; (C) CW2:VO98; (D) CW2:VO78:VE20, Figure S2: Frequency sweep test results: (A) 12HSA2:VO96:VE2; (B) 12HSA2:VO78:VE20; (C) CW2:VO96:VE2; (D) CW2:VO78:VE20.
