# Supplementary material for: Influence of the Mixtures of Vegetable Oil and Vitamin E over the Microstructure and Rheology of Organogels
Source: Gels. 2022 Jan 5;8(1):36. doi: 10.3390/gels8010036 (PMC8774424; doi:10.3390/gels8010036)
Supplement: Supplementary file 1 [file gels-08-00036-s001.zip › gels-1514081-supplementary.pdf]

gels

MDPI

Article

Influence of the Mixtures of Vegetable Oil and Vitamin E over the Microstructure and Rheology of Organogels

Renata Miliani Martinez [1,*], Pedro Leonidas Oseliero Filho [2], Barbara Bianca Gerbelli [3], Wagner Vidal Magalhães [4], Maria Valéria Robles Velasco [1], Suzana Caetano da Silva Lannes [5], Cristiano Luis Pinto de Oliveira [6], Catarina Rosado [7] and André Rolim Baby [1,*]

Supplementary Materials

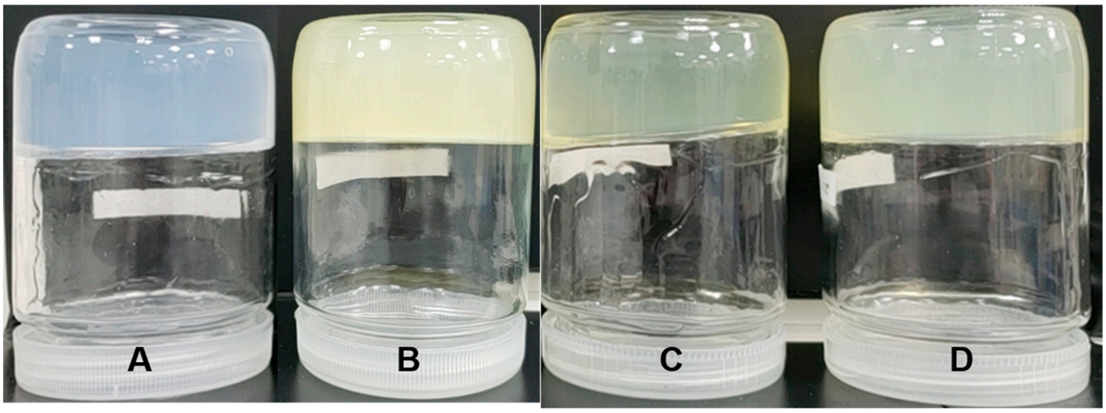

Figure S1. Macroscopic aspect of organogels. (A) 12HSA2:VO98, (B) 12HSA2:VO78:VE20, (C) CW2:VO98, (D) CW2:VO78:VE20.

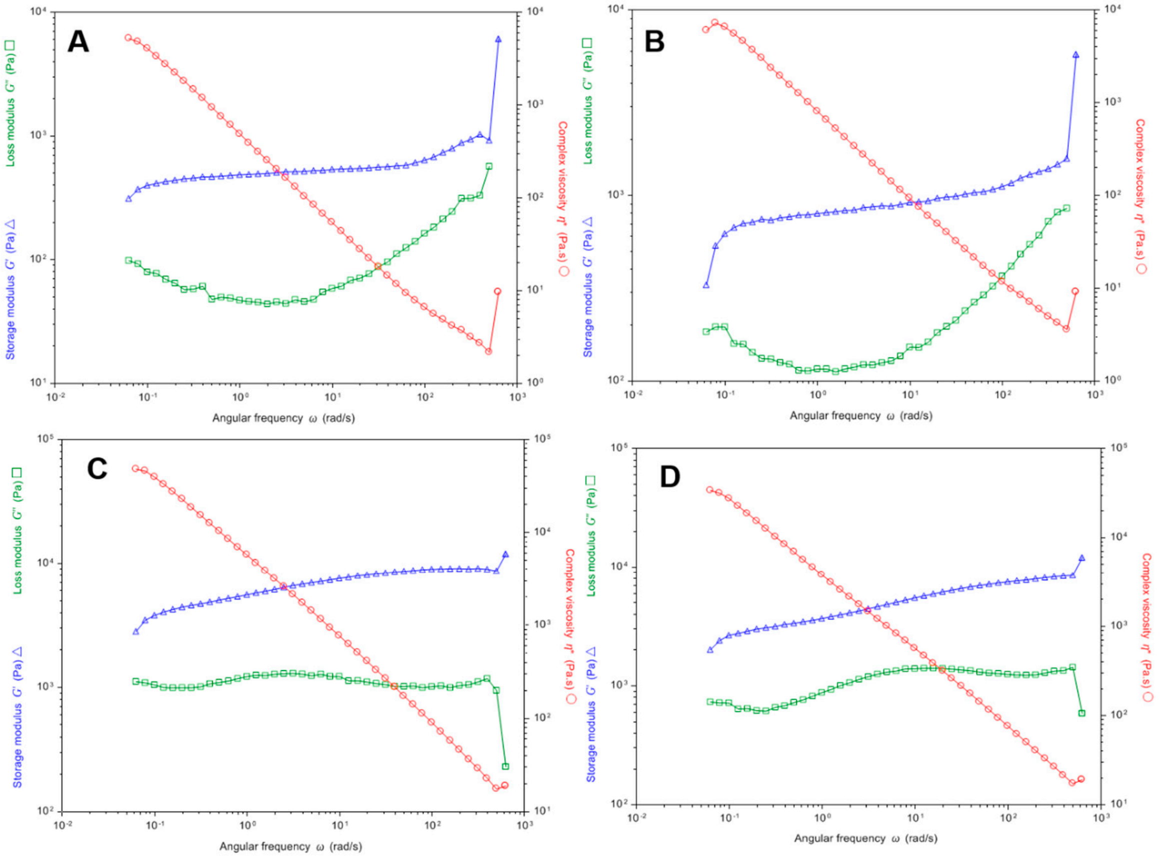

Figure S2. Frequency sweep test. (A) 12HSA2:VO96:VE2, (B) 12HSA2:VO78:VE20, (C) CW2:VO96:VE2, (D) CW2:VO78:VE20.
